# Co‐trimoxazole prophylaxis for children who are HIV‐exposed and uninfected: a systematic review

**DOI:** 10.1002/jia2.26079

**Published:** 2023-06-09

**Authors:** Catherine J. Wedderburn, Ceri Evans, Amy L. Slogrove, Andrea M. Rehman, Diana M. Gibb, Andrew J. Prendergast, Martina Penazzato

**Affiliations:** ^1^ Department of Paediatrics and Child Health and Neuroscience Institute University of Cape Town Cape Town South Africa; ^2^ Medical Research Council Clinical Trials Unit at University College London London UK; ^3^ Department of Clinical Research London School of Hygiene & Tropical Medicine London UK; ^4^ Blizard Institute Queen Mary University of London London UK; ^5^ Zvitambo Institute for Maternal and Child Health Research Harare Zimbabwe; ^6^ Department of Clinical Infection Microbiology and Immunology University of Liverpool Liverpool UK; ^7^ Department of Paediatrics and Child Health Faculty of Medicine & Health Sciences Stellenbosch University Worcester South Africa; ^8^ MRC International Statistics & Epidemiology Group Department of Infectious Disease Epidemiology London School of Hygiene & Tropical Medicine London UK; ^9^ Department of Global HIV Hepatitis and Sexually Transmitted Infections Programmes World Health Organization Geneva Switzerland

**Keywords:** co‐trimoxazole, mortality, morbidity, HIV exposure, infants, public health

## Abstract

**Introduction:**

Co‐trimoxazole prophylaxis is recommended for children born to women with HIV to protect those who acquire HIV from opportunistic infections, severe bacterial infections and malaria. With scale‐up of maternal antiretroviral therapy, most children remain HIV‐exposed uninfected (HEU) and the benefits of universal co‐trimoxazole are uncertain. We assessed the effect of co‐trimoxazole on mortality and morbidity of children who are HEU.

**Methods:**

We performed a systematic review (PROSPERO number: CRD42021215059). We systematically searched MEDLINE, Embase, Cochrane CENTRAL, Global Health, CINAHL Plus, Africa‐Wide Information, SciELO and WHO Global Index Medicus for peer‐reviewed articles from inception to 4th January 2022 without limits. Ongoing randomized controlled trials (RCTs) were identified through registries. We included RCTs reporting mortality or morbidity in children who are HEU receiving co‐trimoxazole versus no prophylaxis/placebo. The risk of bias was assessed using the Cochrane 2.0 tool. Data were summarized using narrative synthesis and findings were stratified by malaria endemicity.

**Results:**

We screened 1257 records and included seven reports from four RCTs. Two trials from Botswana and South Africa of 4067 children who are HEU found no difference in mortality or infectious morbidity in children randomized to co‐trimoxazole prophylaxis started at 2–6 weeks of age compared to those randomized to placebo or no treatment, although event rates were low. Sub‐studies found that antimicrobial resistance was higher in infants receiving co‐trimoxazole. Two trials in Uganda investigating prolonged co‐trimoxazole after breastfeeding cessation showed protection against malaria but no other morbidity or mortality differences. All trials had some concerns or a high risk of bias, which limited the certainty of evidence.

**Discussion:**

Studies show no clinical benefit of co‐trimoxazole prophylaxis in children who are HEU, except to prevent malaria. Potential harms were identified for co‐trimoxazole prophylaxis leading to antimicrobial resistance. The trials in non‐malarial regions were conducted in populations with low mortality potentially reducing generalizability to other settings.

**Conclusions:**

In low‐mortality settings with few HIV transmissions and well‐performing early infant diagnosis and treatment programmes, universal co‐trimoxazole may not be required.

## 1 INTRODUCTION

Vertical transmission of HIV has declined enormously over recent years due to the widespread scale‐up of antiretroviral therapy (ART) among pregnant and breastfeeding women. However, despite this marked progress, there were an estimated 150,000 new HIV infections among children in 2020 [1]. Mortality in children with HIV not receiving ART is around 50% by 2 years of age, with the highest mortality between 2 and 3 months [2, 3]. Co‐trimoxazole is a combination antibiotic of trimethoprim and sulfamethoxazole that is inexpensive, well‐tolerated and effective in preventing opportunistic infections, severe bacterial infections and malaria among infants and children living with HIV [4, 5, 6, 7].

In 2000, the World Health Organization (WHO) first recommended the use of co‐trimoxazole for infants born to women with HIV in sub‐Saharan Africa, regardless of infant HIV status [8]. This recognized that where early infant diagnosis is delayed or unavailable, and where the ongoing risk of postnatal HIV transmission through breastfeeding exists, it was pragmatic for all infants who are HIV‐exposed to receive co‐trimoxazole, to reduce morbidity and mortality in those who acquire HIV [9]. In 2020, approximately 40% of children with HIV remained undiagnosed, and access to early infant diagnosis was estimated at 63% among HIV‐exposed infants by 2 months of age [1]. There are also delays in the testing‐to‐treatment continuum in many settings. While point‐of‐care testing can improve results turnaround time, many countries continue to rely on testing samples at centralized laboratories using conventional DNA PCR which have been estimated to take 35–45 days [10, 11]. In a recent meta‐analysis, 33% of HIV‐exposed infants did not have access to point‐of‐care tests, and only 52% of infants with HIV had initiated ART by 60 days using standard‐of‐care testing [10]. As a result of gaps in the prevention and identification of children with HIV, the guidelines continued to recommend co‐trimoxazole prophylaxis for all HIV‐exposed infants [12].

However, since the majority of children exposed to HIV now remain uninfected [1, 13], it is important to understand if there are benefits of co‐trimoxazole prophylaxis in this expanding population. Although children who are HIV‐exposed and uninfected (HEU) have health and development disparities compared to children who are HIV‐unexposed [14, 15, 16, 17], there is uncertainty over the risks and benefits of co‐trimoxazole prophylaxis in children who are HEU and concerns have been raised over the potential promotion of antimicrobial resistance and microbiome dysbiosis [18, 19, 20]. A previous systematic review conducted in 2006 found no trials of co‐trimoxazole prophylaxis in children who are HEU [7]; however, new trials have been conducted since. The aim of this systematic review was to assess the effect of co‐trimoxazole prophylaxis on morbidity and mortality in children who are HEU to inform the 2021 revision of WHO consolidated HIV guidelines.

## 2 METHODS

### 2.1 Protocol and registration

This systematic review was performed in accordance with the Preferred Reporting Items for Systematic Review and Meta‐Analyses (PRISMA) guidelines and the search strategy was pre‐registered on the International Prospective Register of Systemic Reviews (PROSPERO; CRD42021215059) (Appendix S1).

### 2.2 Search strategy and inclusion criteria

We systematically searched OvidSP MEDLINE, OvidSP Embase, Wiley Cochrane CENTRAL Database (Cochrane HIV/AIDS Trials Registry), OvidSP Global Health, Ebsco CINAHL Plus, Ebsco Africa‐Wide Information, SciELO and WHO Global Index Medicus. Our original search was from inception to 27th January 2021 without language limits; the search was repeated on 4th January 2022 and these updated results are presented. We also searched for ongoing randomized controlled trials (RCTs) through clinicaltrials.gov and the WHO International Clinical Trials Registry Platform and for relevant conference abstracts from the International AIDS Society, the International Workshop on HIV & Pediatrics, the Workshop on Children and Adolescents with Perinatal HIV Exposure, and the Conference on Retroviruses and Opportunistic Infections from the past 5 years. Reference lists of eligible studies and review articles were hand‐searched and selected experts in the field were contacted to identify any additional unpublished research. The full search strategy is shown in Appendix S2. All search terms related to the search concepts “HIV/human immunodeficiency virus,” “infant/child” and “cotrimoxazole/trimethoprim‐ sulfamethoxazole” were used and adapted for entry into electronic databases, combined with database‐specific filters where available. MeSH headings were used in addition to keywords in MEDLINE and Emtree terms in Embase.

We included RCTs that examined the use of co‐trimoxazole prophylaxis given at any dose, duration, frequency or formulation (intervention) compared to placebo or no treatment (control) among children who are HEU (population; defined as children born to a woman with HIV but confirmed as HIV negative by a nucleic acid or antibody test according to infant age; further details in Appendix S3) [21]. Since WHO recommends starting co‐trimoxazole prophylaxis at 4–6 weeks of age, our primary research question examined starting co‐trimoxazole prophylaxis or not in infancy (first year after birth) per current recommendations; where studies examined the effects of extended co‐trimoxazole prophylaxis after cessation of breastfeeding we included these as a secondary question.

Inclusion criteria: trials had to be published in a peer‐reviewed journal and examine one or more of the following outcomes, separately or as a composite: (1) all‐cause mortality; (2) all‐cause hospitalization; (3) infectious morbidity, including but not limited to diarrhoea, pneumonia, *Pneumocystis jirovecii* pneumonia (formerly *Pneumocystis carinii* pneumonia; PCP), tuberculosis, malaria, toxoplasmosis and other parasitic infections, severe bacterial infections; (4) treatment‐related adverse events and serious adverse events (including neutropenia, anaemia and other haematological abnormalities); (5) child growth and development; or (6) antimicrobial resistance and microbiome. Studies were limited to humans. We excluded studies that examined children with HIV or with unknown HIV status.

Citations from all databases were transferred to EndNote and de‐duplicated separately by two authors (CJW and CE), who then screened the remaining titles and abstracts independently for inclusion; any conflicts in article selection were resolved through mutual discussion. Full‐text articles were obtained for all selected abstracts meeting the eligibility criteria and two reviewers (CJW and CE) assessed each full‐text article for eligibility to determine the final study selection. Differences were resolved through a third reviewer (AJP).

### 2.3 Data extraction

Study characteristics and data including population demographics, intervention, follow‐up and pre‐specified outcomes were extracted from studies meeting the inclusion criteria independently by two reviewers (CJW and CE). Study investigators were contacted for unreported data and additional details where necessary. The risk of bias was assessed using the Cochrane Collaboration's Risk of Bias 2.0 tool [22] by the same two independent reviewers. Methodological components of the studies were assessed and classified as high‐risk, low‐risk or some concerns of bias. Any discrepancies were discussed and clarified with other authors (ALS, AJP and MP) to make a final recommendation.

### 2.4 Data analysis

We first performed a narrative synthesis of data from eligible studies. Data were displayed by outcome measure; measures of effect were included as reported by the individual studies. Crude and adjusted results were included using the adjustments outlined in each study. Outcomes were assessed as they had been analysed using intention‐to‐treat principles. We stratified by age at randomization as per the primary and secondary research questions and by malaria endemicity (low prevalence/non‐malarial region vs. high prevalence). Where there were studies reporting the same outcomes with the same hypotheses, we planned to do a random‐effects meta‐analysis. We planned to assess statistical heterogeneity using the *χ*2 and *I* ^2^ statistics, and publication bias using a funnel plot where there were at least five trials.

### 2.5 Grading quality of evidence

The GRADE approach was used to assess the overall quality of evidence and recommendations of the systematic review [23]. GRADE tables were created using GRADEPro software and mortality and morbidity outcomes were rated according to the GRADE framework.

## 3 RESULTS

### 3.1 Systematic review findings

**Figure 1:**
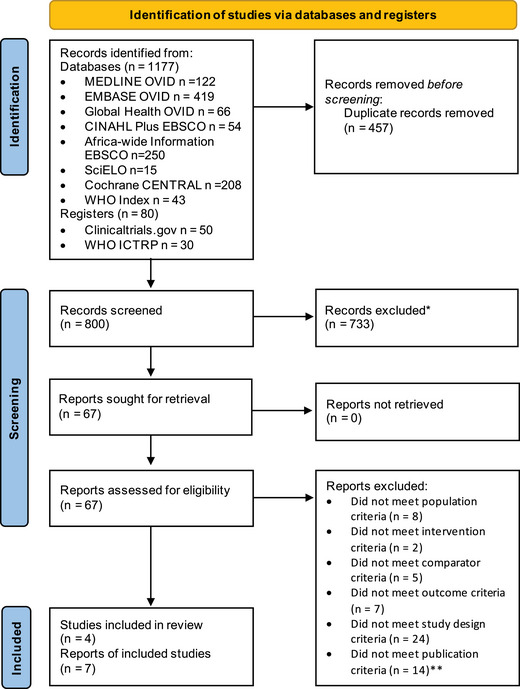
PRISMA flow diagram. PRISMA 2020 flow diagram for new systematic reviews which included searches of databases and registers only. Template accessed from: www.prisma‐statement.org. *Adapted from*: Page MJ, McKenzie JE, Bossuyt PM, Boutron I, Hoffmann TC, Mulrow CD, et al. The PRISMA 2020 statement: an updated guideline for reporting systematic reviews. BMJ 2021;372:n71. https://doi.org/10.1136/bmj.n71. *Reasons for exclusion were primarily population criteria (children living with HIV instead of children who were HIV‐exposed and uninfected) or design criteria (studies were not randomized controlled trials). **For all conference abstracts, we found and checked the equivalent full‐text article.

We identified 1257 records, from database searches (*n* = 1177) and trial registers (*n* = 80). After removing duplicates and screening for exclusions (Appendix S4), 67 full‐text articles were assessed for eligibility. The narrative synthesis included seven reports from four RCTs [24, 25, 26, 27, 28, 29, 30] (Figure 1; Appendix S5). Two trials (contributing two reports each) from Botswana and South Africa examined the use of co‐trimoxazole from early infancy according to current guidelines [24, 25]. Two trials (three reports) examined the continuation of co‐trimoxazole prophylaxis after cessation of breastfeeding in Uganda [28, 29, 30]. Our updated search identified no additional trials compared to the original search.

**Figure 2:**
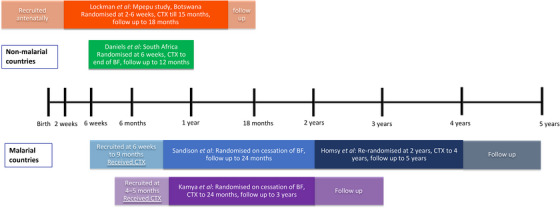
Synthesis of included studies. Abbreviations: BF, breastfeeding; CTX, co‐trimoxazole prophylaxis. Notes: Daniels et al.: Duration of breastfeeding over 12 months: 50% with co‐trimoxazole; 47% with no co‐trimoxazole breastfed; median duration not reported. Sandison et al.: Median age at randomization 9.6 months (IQR 8.3–12.4) in co‐trimoxazole continued group versus 10.0 months (8.9–13.5) in the co‐trimoxazole stopped group. Kamya et al.: Median age at randomization 11.6 months (6.2–18.7) in the co‐trimoxazole group versus 10.0 months (6.0–18.0) in the no co‐trimoxazole group.

Details of the trials are provided in Table 1 and Figure 2. The Botswana trial (MPEPU) [24] (*n* = 2848) considered whether co‐trimoxazole was superior to placebo, starting from 14 to 34 days of age and continuing to 15 months of age. Mortality was assessed up to 18 months. Among the 20% of breastfed children, this trial had a second randomization to either 6 or 12 months of breastfeeding. The South African trial [25] (*n* = 1219) was a non‐inferiority trial of co‐trimoxazole from 6 weeks of age for the duration of breastfeeding, compared to no prophylaxis (non‐inferiority margin: absolute increase of up to 5% [and relative increase of 1.7] in the composite outcome of grade 3 and 4 diarrhoea/pneumonia or mortality to 12 months), and was not blinded to participants, but clinical and laboratory staff were masked to group assignment. Overall, 50% of children in the co‐trimoxazole group and 47% in the no co‐trimoxazole group breastfed for over 12 months. Outcomes were assessed at age 12 months. In the two Ugandan trials conducted in malaria‐endemic settings, all infants initiated co‐trimoxazole prophylaxis at age 4–6 weeks and were then randomized to continue prophylaxis or not after cessation of breastfeeding. In one of the trials (Sanderson et al.), 185 infants were randomized to either continue or discontinue co‐trimoxazole after cessation of breastfeeding until 24 months of age [28]; those who had co‐trimoxazole to 24 months (*n* = 91) were then re‐randomized to continue co‐trimoxazole to age 4 years versus discontinue (Homsy et al.) [29]. Although there is overlap, we treated the reports as separate studies in one trial with individual risk of bias assessments and we report the results for each randomization time period separately. In the other study (Kamya et al.), infants were randomized at the end of breastfeeding to either stop or continue co‐trimoxazole to 24 months (*n* = 93), or to two other antimalarial regimens [30].

**Table 1:** Trials of co‐trimoxazole prophylaxis meeting inclusion criteria.

					*N* enrolled by randomization group		
Study	Country and enrolment dates	Population	Study type	Intervention	Co‐trimoxazole	No co‐trimoxazole	Study primary outcome
(Daniels et al.) [25]	South Africa 2013–2018	Children recruited at 6 weeks from PMTCT programmes in two clinics; randomized to intervention from 6 weeks until the end of breastfeeding (6 weeks after cessation). Infants with birthweight <2 kg excluded.	Randomized controlled non‐inferiority trial^a^ Single blinded (clinical team/laboratory masked)	Co‐trimoxazole versus no prophylaxis	611	609^b^	Combined grade 3 or 4 common childhood illnesses (pneumonia and diarrhoea) or all‐cause mortality by 12 months
(D'Souza et al.) [27]	South Africa *2013‐2018*	163 stool samples from 63 HEU infants from Daniels et al., 2019	Sub‐study	Co‐trimoxazole versus no prophylaxis	34	29	Gut microbiota profiles: alpha & beta diversity, and resistance gene carriage by shotgun metagenomic sequencing
(Lockman et al.) [24]	Botswana (Mpepu study) 2011–2015	Women recruited in pregnancy from antenatal clinics/maternal wards in a city, town and village; children randomized to intervention or placebo from 14 to 34 days until 15 months.	Randomized controlled double‐blinded superiority trial	Co‐trimoxazole versus placebo; second randomization: breastfed children to 6 or 12 months breastfeeding	1423	1425	Cumulative mortality from treatment assignment to age 18 months (and HIV‐free survival by breastfeeding duration)
(Powis et al.) [26]	Botswana *2011‐2015*	381 stool samples from 220 infants from Lockman et al., 2017	Sub‐study	Co‐trimoxazole versus placebo	105	115	Bacterial resistance in *E. coli* and *Klebsiella* using disc diffusion methods
(Sandison et al.) [28]	Uganda 2007–2008	Children enrolled aged between 6 weeks and 9 months from a maternal child health clinic; randomized after cessation of breastfeeding (median 9.6 months intervention, 10 months control) to intervention until 24 months.	Superiority randomized controlled trial; Not blinded	Co‐trimoxazole versus no prophylaxis	98	87	Malaria incidence at 24 months
(Homsy et al.) [29]	Uganda *2007‐2008*	Children from Sandison et al., 2011 who received intervention to 24 months randomized again to intervention until 48 months. Follow up till 60 months. Included observational child HEU cohort.	Superiority randomized controlled trial; Not blinded	Co‐trimoxazole versus no prophylaxis	45	46	Malaria incidence at 48 months
(Kamya et al.) [30]	Uganda 2010–2011	Children enrolled aged 4–5 months from a maternal child health clinic randomized to one of four regimens after cessation of breastfeeding (median age 10 months; control 10.0; co‐trimoxazole 11.6 months) to 24 months with a follow up to 36 months.	Superiority randomized controlled trial; Not blinded	Daily co‐trimoxazole versus no prophylaxis versus monthly sulfadoxine‐pyrimethamine versus dihydoartemisinin‐piperaquine	47	46	Malaria incidence at 24 months

All intention‐to‐treat analyses.

^a^ Non‐inferiority margin: absolute increase of up to 5% (and relative increase of 1.7) in composite outcome.

^b^ 608 controls analysed in intention‐to‐treat as one infant excluded for receiving traditional medicine.

Lockman et al. and Daniels et al. co‐trimoxazole dose: 100 mg sulfamethoxazole/20 mg trimethoprim to 6 months or weight <5 kg; 200 mg sulfamethoxazole/40 mg trimethoprim after 6 months or weight >5 kg; Sandison et al.: <15 kg (200 mg/40 mg 5 ml suspension: <= 4 kg 2.5 ml/day, 4–8 kg 5 ml/day, 8–15 kg 10 ml/day); 10–15 kg (400 mg/80 mg).

Breastfeeding:

Daniels et al. after 12 months: co‐trimoxazole group 50%; no co‐trimoxazole group 47%.

Lockman et al.: co‐trimoxazole group 142 (10%) for 6 months, 144 (10%) to 12 months; placebo 138 (10%) to 6 months, 145 (10%) to 12 months.

Risk of bias assessment judged 2/5 (40%) studies as having a high risk of bias and 3/5 (60%) having some concerns regarding bias (Appendix S6). Both studies addressing the primary research question had fewer children than planned in the final analysis. In the trial in Botswana, only 58% (1742 out of 3016 planned) completed the 18‐month study visit due to early trial closure on advice of the data and safety monitoring board (DSMB). In the South African trial, only 65% (841 out of 1298 planned) provided 12‐month study visit data due to loss to follow‐up and early study closure; it was not possible to ascertain whether those infants lost to follow‐up had died or been admitted to hospital. Maternal CD4 cell count differed between groups (lower CD4 in the co‐trimoxazole group), although adjusted analyses were performed. Separate reports from these trials examined antimicrobial resistance in a subgroup of children (*n* = 220 [Botswana] and *n* = 63 [South Africa]) but sample selection criteria were unclear. Finally, none of the studies in Uganda were blinded with potential for detection bias and there were concerns over selection and attrition bias in one [29]; overall sample sizes were smaller (ranging from 91 to 185), and these trials addressed the question of stopping co‐trimoxazole prophylaxis rather than starting.

For the primary question of providing co‐trimoxazole prophylaxis or not in infancy, there was no evidence of a difference in mortality between co‐trimoxazole versus control groups in either the trial from Botswana (30 deaths co‐trimoxazole vs. 34 placebo; estimated mortality 2.4% vs. 2.6%) [24] or South Africa (2 co‐trimoxazole vs. 1 no prophylaxis; absolute mortality 0.3% vs. 0.2%) [25]. Similarly, the trials found no evidence of benefit of co‐trimoxazole for pneumonia, diarrhoea or a composite outcome of these with mortality, and hospitalizations in the Botswanan trial (Table 2). The Botswanan trial separately found similar hospitalizations between groups (12.5% co‐trimoxazole vs. 17.4% placebo group; difference –4.9% [95% CI –12.2, 2.4], *p* = 0.19). In the South African study, the primary analysis was to assess the non‐inferiority of the composite outcome; the authors reported that no co‐trimoxazole prophylaxis was non‐inferior and not superior to prophylaxis [25, 31].

**Table 2:** Mortality and morbidity results of trials examining the effects of co‐trimoxazole prophylaxis on children who are HEU.

Study	Mortality (deaths)			Hospitalization			Diarrhoea			Pneumonia			Composite outcome			Malaria (episodes)		
	CTX+	CTX−	*p*‐value	CTX+	CTX−	*p*‐value	CTX+	CTX−	*p*‐value	CTX+	CTX−	*p*‐value	CTX+	CTX−	*p*‐value	CTX+	CTX−	*p*‐value
(Daniels et al.) [25]	2 (<1%)	1 (<1%)	*p* = 0.584 at day 400	−	−	−	12 (2%)	11 (2%)	*p* = 0.888	38 (6%)	27 (4%)	*p* = 0.204	49 (8%)	39 (6%)	*p* = 0.333 HR 1.23 (0.81−1.87)	−	−	−
(Lockman et al.) [24]	30 (2.4%)	34 (2.6%)	*p* = 0.70 at 18 months Difference: −0.2% (−1.5 to 1.0)	125 (12.5%)	143 (17.4%)	*p* = 0.19 Difference: −4.9% (−12.2 to 2.4)	10.3%	9.0%	*p* = 0.26	2.5%	3.6%	*p* = 0.33	212 (17.3%)	237 (19.6%)	*p* = 0.13 Difference: −2.2% (−5.2 to 0.7)	−	−	−
(Sandison et al.) [28]	2	1	*p* = 0.61 at 24 months	8	4	*p* = 0.79	189 (1.83 ppy)	170 (1.96 ppy)	*p* = 0.48 aIRR: 0.91 (0.70−1.18)	33 (0.32 ppy)	20 (0.23 ppy)	*p* = 0.27 aIRR 1.37 (0.78−2.40)	−	−	−	299 (3.24 ppy)	400 (5.57 ppy)	*p* = 0.001 aIRR 0.61 (0.46–0.81)
(Homsy et al.) [29]	0	0	No significant difference	15 (0.180 ppy)	17 (0.189 ppy)	No significant difference	40 (0.48 ppy)	36 (0.40 ppy)	No significant difference	14 (0.168 ppy)	6 (0.067 ppy)	No significant difference	−	−	−	243 (2.91 ppy)	503 (5.60 ppy)	*p*<0.0001 aIRR 0.53 (0.39–0.71)
(Kamya et al.) [30]	2	2	−	8 (0.178 ppy)	10 (0.211 ppy)	*p* = 0.69 PE: 21% (−147 to 75)	98 (2.18 ppy)	101 (2.13 ppy)	*p* = 0.91 PE: −2% (−53 to 32)	158 (3.52 ppy)	146 (3.08 ppy)	*p* = 0.65 PE: −8% (−52 to 23)	−	−	−	116 (2.86 ppy)	240 (6.28 ppy)	*p* = 0.001 Adjusted PE: 49% (23−66)

Abbreviations: aIRR, adjusted incidence rate ratio; CTX, co‐trimoxazole; HR, hazard ratio; PE, protective efficacy; ppy, incidence per person year at risk; SOC, standard of care.

Definition of composite outcome:

Daniels et al.: incidence of grade 3 and 4 pneumonia or diarrhoea or all‐cause mortality until 12 months of age.

Lockman et al.: death, admission to hospital or grade 3–4 clinical adverse events at 18 months.

Additional information: Daniels et al.: composite outcome adjusted for maternal CD4 aHR 1.25 (95% CI 0.80–1.97)**;** risk difference (CTX− minus CTX+): −0.0319 (−0.075 to 0.011); upper 95% CI did not reach non‐inferiority boundary.

Lockman et al.: percentages are presented as estimated proportions at age 18 months; hospitalization represents ≥1 admission to hospital.

Sandison et al.: adjusted analyses adjusted for age at randomization.

Homsy et al.: we present data from the second randomization only (HEU children randomized to continue or stop co‐trimoxazole at 2 years). Significance was assessed by comparison to a standard of care arm (HEU children stopping co‐trimoxazole after cessation of breastfeeding).

Kamya et al.: adjusted analyses accounted for age at randomization and incidence of malaria prior to randomization.

Further, there was little evidence for a difference in growth faltering, although this was defined as either underweight or stunting at 6 or 12 months combined (19.7% co‐trimoxazole vs. 23.8% no co‐trimoxazole, *p* = 0.08), and it was not possible to disaggregate these growth outcomes at each age from the data presented. Overall, co‐trimoxazole prophylaxis appeared to be safe, and there was no difference in clinical adverse events or anaemia prevalence between arms in both studies. However, neutropenia, defined using DAIDS grading [32], was more frequent in the co‐trimoxazole group in the Botswanan study (8.1% vs. 5.8%, *p* = 0.03) [24] (Appendix S7).

Neither trial reported on PCP, tuberculosis, parasitic infections or child neurodevelopment. However, smaller sub‐studies of the two trials reported bacterial antimicrobial resistance and analysis of the infant gut microbiome while taking co‐trimoxazole. In Botswana, the proportion of participants with *Escherichia coli* (*E. coli*) resistance to co‐trimoxazole isolated from stool samples was higher in the co‐trimoxazole versus placebo group at 3 months (95% vs. 51%, *p* = 0.001) and 6 months (84% vs. 58%, *p* = 0.01), although baseline resistance was already high at 2–4 weeks of age before co‐trimoxazole was commenced (64.7% co‐trimoxazole arm vs. 60.7% placebo arm). There was also increased *E. coli* resistance to amoxicillin in the co‐trimoxazole group at 3 months (*p* = 0.02) and 6 months (*p* = 0.07), although at baseline, resistance was higher in the placebo group (71.4% vs. 58.8%). Similarly, *Klebsiella* resistance was also higher at 3 months (79% vs. 19%; *n* = 40) and 6 months (69% vs. 14%; *n* = 37) in the co‐trimoxazole versus placebo group. In South Africa, metagenomic analysis of stool samples from infants randomized to co‐trimoxazole (*N* = 34) or no co‐trimoxazole (*N* = 29) showed no significant differences in microbial taxa or functional pathways as assessed by α‐diversity, but an increase in antibiotic resistance gene carriage and diversity in the co‐trimoxazole trial arm at 4 and 6 months, while gut microbiome β‐diversity decreased [27].

For the secondary question of continuing co‐trimoxazole after cessation of breastfeeding, the studies from Uganda found that co‐trimoxazole prophylaxis was protective against malaria through 5 years of age, reducing the incidence of malaria by 39–49% [28, 29, 30]. No effects were seen on mortality, hospitalization, diarrhoea or pneumonia (Table 2). Malnutrition was also reported to be similar across groups in one study [30]. Of note, there were no significant differences in serious adverse events [29, 30] or in markers of antifolate resistance measured in one study, although a high proportion of dhfr/dhps quintuple mutants were found in both groups [28].

### 3.2 Statistical pooling

Statistical pooling was not possible due to the different timing of outcomes reported, concerns over risk of bias and varying co‐trimoxazole exposure duration, therefore, we present the results in narrative form only.

### 3.3 Assessment of evidence quality

**Figure 3:**
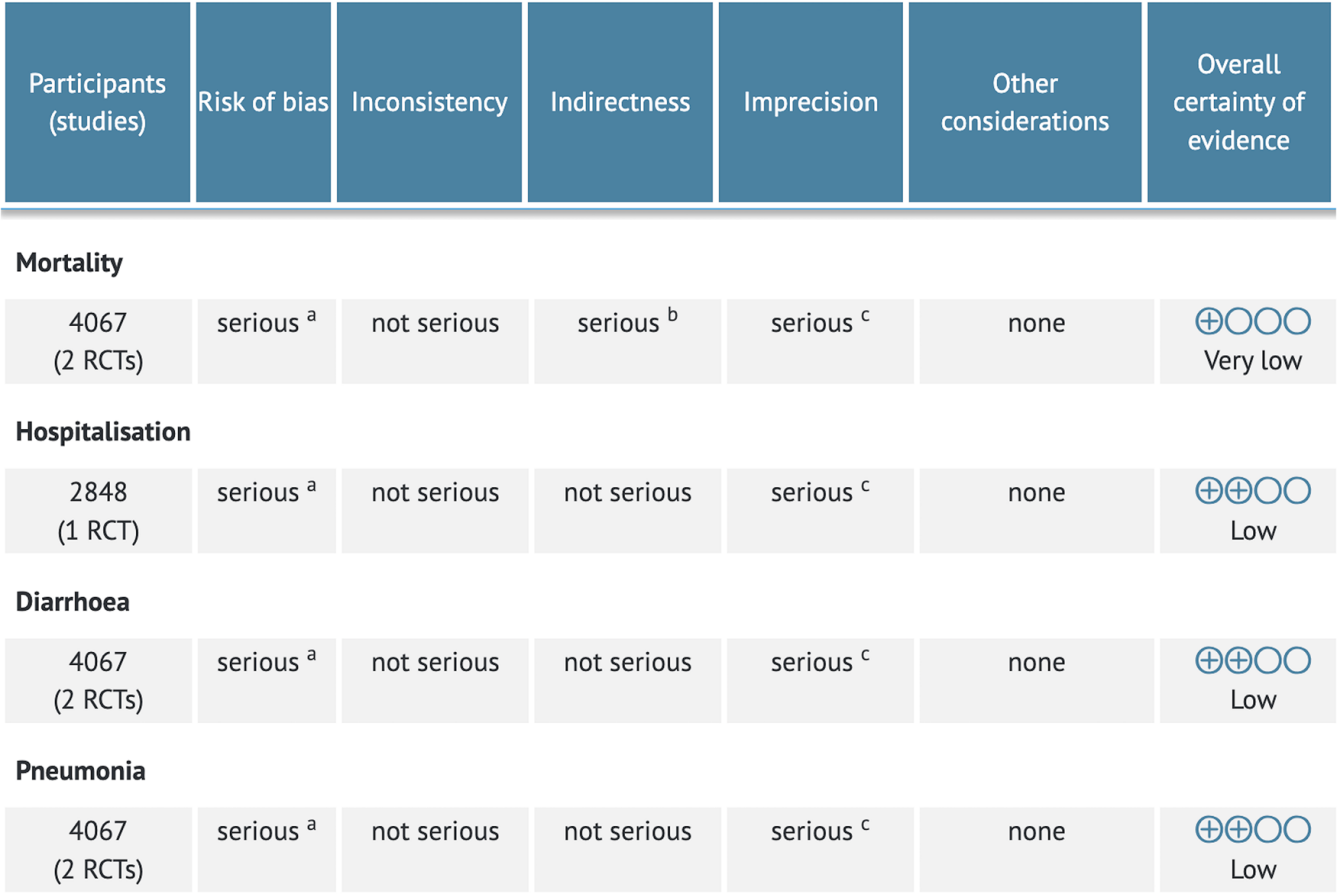
GRADE assessment including studies examining the primary research question. The goal of this GRADE table was to assess the primary question of whether co‐trimoxazole prophylaxis started in infancy impacts morbidity and mortality of children who are HIV‐exposed and uninfected. Therefore, due to indirectness, we did not include the Ugandan studies here. ^a^Risk of bias: some concerns to high risk; ^b^Indirectness: low mortality and unclear generalizability to other LMIC settings. ^c^Imprecision: both trials did not reach their calculated sample size due to stopping early. Figure created using GRADEPRO software (https://gradepro.org/).

We performed a GRADE assessment of the evidence for co‐trimoxazole prophylaxis in early infancy (Figure 3). Overall, there was a very low quality of evidence for mortality, and low quality of evidence for morbidity outcomes. This was due to (1) risk of bias; (2) indirectness, given the low‐mortality settings; (3) unclear generalizability to other low‐ and middle‐ income country settings given low breastfeeding rates in one study; and (4) potential imprecision as the trials did not reach their *a priori* calculated sample sizes and had few mortality events.

## 4 DISCUSSION

Co‐trimoxazole prophylaxis was originally recommended for all infants born to women with HIV starting at 6 weeks of age, until HIV infection had been excluded and the risk of exposure had ceased [8]. The origins of this recommendation were primarily to prevent mortality among infants living with HIV before a diagnosis is possible. This guidance has continued while potential risks and benefits to children who are HEU were being investigated [12, 33]. Accumulating evidence over recent decades has shown that, on average, children who are HEU have more infectious morbidity and mortality compared to children who are HIV‐unexposed [15, 34, 35], particularly when mothers have advanced HIV disease and late ART initiation [35]. This has led to growing interest in understanding the effectiveness of co‐trimoxazole prophylaxis in improving outcomes in children who are HEU, particularly in settings where vertical HIV transmission rates are now low, and early infant diagnosis of HIV infection is high with timely ART initiation. This systematic review, therefore, aimed to evaluate the impact of co‐trimoxazole prophylaxis on clinical outcomes in children who are HEU, to inform a potential revision of WHO guidelines. Three main findings emerged. First, two trials of co‐trimoxazole prophylaxis in non‐malarial regions demonstrated no reductions in mortality or infectious morbidity in children who are HEU through 12–18 months of age. Second, co‐trimoxazole prophylaxis continued beyond the end of breastfeeding in Uganda reduced the incidence of malaria through 5 years of age but did not impact all‐cause mortality or other infectious morbidity. Third, the use of co‐trimoxazole prophylaxis can increase antimicrobial resistance, which is a growing global concern.

Several observational studies of co‐trimoxazole prophylaxis among children who are HEU have found reductions in infectious morbidity [36, 37], while others have not [38]; however, only two randomized trials—both from non‐malarial regions—were identified from our search. A placebo‐controlled trial in Botswana found no benefit of co‐trimoxazole prophylaxis on mortality, hospitalization, diarrhoea or pneumonia through 18 months of age, and a South African non‐inferiority trial found that withholding co‐trimoxazole prophylaxis was non‐inferior to providing co‐trimoxazole prophylaxis in children who are HEU for a combined outcome of mortality and grade 3–4 clinical events through 12 months of age. Further, the South African trial reported no significant effect on a composite growth outcome, and subsequent to our search, a sub‐study from the Botswanan trial also reported no overall effect of co‐trimoxazole on growth [39]. The findings of no benefit were consistent across all trial endpoints, and together, these trials provide the only available randomized data regarding the value of early‐life co‐trimoxazole for children who are HEU in sub‐Saharan Africa.

Clinical trials have established the benefits of co‐trimoxazole in children with HIV in reducing mortality and hospitalization [4, 5, 9]. By contrast, the findings from the current review suggest that there is no clinical benefit of co‐trimoxazole for children who are HEU in non‐malarial settings, such as South Africa and Botswana, which have low vertical HIV transmission (4% and 2%, respectively) [1], high uptake of early infant diagnosis (87% and >98%, respectively) [1], and timely early infant diagnosis and ART initiation. Two recent trials of co‐trimoxazole prophylaxis in children without HIV, but with severe acute malnutrition or severe anaemia (some of whom may be HEU), also showed no mortality reductions, despite a lower incidence of malaria and some bacterial infections [40, 41].

Three Ugandan trials assessed a different question: the use of prolonged co‐trimoxazole prophylaxis beyond cessation of breastfeeding. These trials, therefore, did not address the primary question of whether all infants born to women with HIV need to start co‐trimoxazole after birth but provided valuable randomized data on the longer‐term effects of co‐trimoxazole for children who are HEU, particularly in settings of endemic malaria transmission. Overall, the trials found that co‐trimoxazole was protective against malaria through 5 years of age, as summarized in a previous systematic review [42], although there was no evidence of benefit for all‐cause mortality. However, dedicated evidence‐based interventions are available for malaria prevention among all children, including those who are HIV‐exposed. Of note in Kamya et al., monthly dihydroartemisin‐piperaquine had a greater protective efficacy against malaria at 24 months compared to co‐trimoxazole. Malaria control efforts are evolving, particularly given recent findings of the substantially lower incidence of uncomplicated malaria, severe malaria and death from malaria among children receiving sulfadoxine–pyrimethamine and amodiaquine chemoprophylaxis and RTS,S/AS01E malaria vaccine compared to either intervention alone [43].

Concerns over the impact of co‐trimoxazole prophylaxis on antimicrobial resistance have been raised previously [18, 19]. We identified two trial sub‐studies which concluded that the use of co‐trimoxazole prophylaxis leads to increased antimicrobial resistance, measured using disc diffusion methods among *E. coli* and *Klebsiella* isolates in Botswana, or resistance gene carriage using whole‐metagenome shotgun sequencing in South Africa. These findings are consistent with an effect of co‐trimoxazole on resistance genes due to persistent antibiotic selection pressure and this concurs with a prior report from Zambia that found modestly increased co‐trimoxazole and clindamycin resistance among HIV‐exposed infants receiving co‐trimoxazole [44]. However, the background prevalence of co‐trimoxazole resistance is extremely high in these settings. In the Botswana trial, over 60% of infants at 2–4 weeks of age already had *E. coli* isolates which were resistant to co‐trimoxazole. In adults and children with HIV, co‐trimoxazole prophylaxis continues to have clinical benefits despite this high background antimicrobial resistance, and is, therefore, recommended for these populations [45]. Furthermore, in most settings, co‐trimoxazole is not used as a first‐line treatment for intercurrent illnesses. Although the Botswana study found some evidence of increased resistance to amoxicillin which is used as a first‐line treatment for common childhood infections, the baseline prevalence of amoxicillin resistance differed between arms in the few children studied; this needs to be explored further in future studies, as cross‐class resistance would be a major concern. The South African study showed that co‐trimoxazole led to alterations in the infant gut microbiome but did not promote major dysbiosis, since microbial taxa and functional pathways were not substantially different between infants receiving or not receiving co‐trimoxazole at 4 and 6 months. Overall, these findings suggest that caution should be applied; however, the long‐term impact remains uncertain and further studies are needed.

This systematic review has several limitations. There was under‐ascertainment of the primary outcome in the trials in Botswana and South Africa, with 58% and 65% of the planned sample size in each trial, respectively. Further, there was lower than anticipated mortality among the child HEU populations in both the Botswanan trial (2.5%) and South African trial (<1%) compared to estimates of child HEU mortality derived from prior studies (5.2% [46] and 6.7% [47]) which the sample size calculations were based upon. This may have reduced the statistical power to detect mortality differences. Further, it raises concerns over the representativeness of the study populations and the corresponding generalizability of translating these trial results to settings with higher mortality as well as epidemic contexts with higher vertical HIV transmission rates and greater burdens of severe bacterial infection. While the trial in Botswana reported more neutropenia in the group receiving co‐trimoxazole, this was assessed using DAIDS grading [32] without adapting cut‐offs for African children, who have lower neutrophil counts [48, 49, 50]; therefore, neutropenia may have been over‐estimated. Additionally, given the study censorship, the median duration of co‐trimoxazole prophylaxis is difficult to fully ascertain for each trial. There was at least some concern of bias across all included studies, and the small number of studies identified and differences in methodology precluded meta‐analysis. Although similar findings across trials strengthen the conclusion, the identified risk of bias and imprecision impacted the certainty of evidence.

Further evidence is needed on co‐trimoxazole prophylaxis in settings with higher infant mortality and vertical HIV transmission. Recently, the external validity of morbidity and mortality rates in RCTs has been questioned due to the standard of care offered, and hospitalization rates were noted to decrease in children participating in the Botswanan RCT [51]. These findings support operational research to investigate the real‐world impact of different co‐trimoxazole strategies in countries with and without mature HIV programmes. Currently, co‐trimoxazole prophylaxis is not recommended for neonates due to theoretical concerns regarding kernicterus [52]. Given that peak mortality in HIV‐exposed infants occurs in the first few months after birth [2, 3], future work needs to be done to investigate interventions earlier in life. However, strengthening early infant diagnosis programmes and expanding the use of point‐of‐care testing and early ART initiation in parallel with optimum maternal HIV care should remain the focus of HIV programmes. The clinical relevance of the antibiotic resistance and microbiome changes also needs to be determined, and we need more information on child growth and neurodevelopment to comprehensively understand early‐life impacts on children who are HEU [53, 54].

## 5 CONCLUSIONS

In conclusion, this review aimed to examine the risks and benefits of the current policy of providing co‐trimoxazole from 4 to 6 weeks of age to all children who are HIV‐exposed. This review provides evidence that in non‐malarial settings with low mortality, there is no clinical benefit of co‐trimoxazole prophylaxis for children who are HEU. These findings were shared with a WHO‐convened technical working group and informed the 2021 revision of the WHO Consolidated HIV guidelines [45], which now include specific implementation considerations for settings similar to those where the trials were undertaken. Questions remain for other countries with high infant mortality and morbidity, and outside of trial settings where standard‐of‐care may be less optimal, to inform new approaches to closing the health gap between children who are HEU and those who are HIV‐unexposed.

## COMPETING INTERESTS

AJP declares paid participation on the Botnar Research Centre for Child Health independent external review board. He is a member of several DSMBs with no payment, none of which relate to the current research. All other authors declare no competing interests.

## AUTHORS’ CONTRIBUTIONS

CJW and MP conceived the study. CJW, AJP and MP wrote the protocol with contributions from CE, ALS and AMR. CJW performed the literature search. CJW and CE screened and reviewed articles, extracted and synthesized the data. ALS, AMR, DMG, AJP and MP provided critical input. CJW, CE and AJP drafted the first iteration of the manuscript. ALS, AMR, DMG and MP reviewed and revised the manuscript for intellectual content.

## FUNDING

World Health Organization. CE and AJP are supported by Wellcome [210807/Z/18/Z and 108065/Z/15/Z, respectively]. AMR is partially funded by the UK Medical Research Council (MRC) and the UK Department for International Development (DFID) under the MRC/DFID Concordat agreement which is also part of the EDCTP2 programme supported by the MRC [MR/R010161/1]. ALS is supported by the Fogarty International Center of the National Institutes of Health under award number 1K43TW010683.

## Supporting information

Supporting information

## Data Availability

Protocol and materials are available on PROSPERO and in Supplementary information.
