# Supplementary material for: Co‐trimoxazole prophylaxis for children who are HIV‐exposed and uninfected: a systematic review
Source: J Int AIDS Soc. 2023 Jun 9;26(6):e26079. doi: 10.1002/jia2.26079 (PMC10251133; doi:10.1002/jia2.26079)
Supplement: Supplementary file 1 — Supporting information [file JIA2-26-e26079-s001.docx]

**Supplementary information**

**Co-trimoxazole prophylaxis for children who are HIV-exposed and uninfected:**

**a systematic review**

Catherine J Wedderburn, Ceri Evans, Amy L Slogrove, Andrea M Rehman, Diana M Gibb, Andrew J Prendergast*, Martina Penazzato*

*Joint senior authors

**S1 Appendix:** PROSPERO systematic review protocol

**S2 Appendix:** Electronic search strategy by database

**S3 Appendix:** Definition of children who are HIV-exposed and uninfected

**S4 Appendix:** Characteristics of excluded studies on full-text review

**S5 Appendix:** Details of included trials

**S6 Appendix:** Risk of bias assessments

**S7 Appendix:** Neutropenia and anaemia in trials examining the effects of co-trimoxazole prophylaxis

on children who are HEU

**S1 Appendix: PROSPERO systematic review protocol**

**Link:** <https://www.crd.york.ac.uk/prospero/display_record.php?ID=CRD42021215059>

**Protocol deviations:**

- We planned to present the trial results by infant sex, maternal and infant ART exposure. However, trial results were not presented stratified by these characteristics and therefore it was not possible to do this. In some cases infant sex was not reported (Sandison *et al;* Lockman *et al*) and in others, maternal ART was not reported (Sandison *et al,* Homsy *et al,* Kamya *et al)*

**S2 Appendix**

**Electronic search strategy (by database)**

**OvidSP MEDLINE search**

- 1. (HIV or HIV-1 or HIV-2 or human immunodeficiency virus or human immune-deficiency virus or human immuno-deficiency virus or AIDS or Acquired Immunodeficiency Syndrome or Acquired Immune-Deficiency Syndrome or acquired immunedeficiency syndrome or acquired immuno-deficiency syndrome or HEU or PMTCT or mother-to-child transmission or vertical transmission or maternal-fetal transmission or maternal-foetal transmission or fetomaternal Infection or maternal-fetal infection or maternal-foetal infection or vertical infection).mp
  2. exp HIV/ or exp HIV-2/ or exp HIV-1/ or exp HIV INFECTIONS/
  3. 1 or 2
  4. (infan* or newborn* or new-born* or perinat* or neonat* or neo-nat* or preemie* or baby or babies or toddler* or child* or pediatric* or paediatric* or girl* or boy* or preschool* or pre-school*).mp
  5. (("1" or "2" or "3" or "4" or "5" or "6" or "7" or "8" or "9" or "10" or "11" or "12" or "13" or "14" or "15" or "16" or "17" or "18" or "19" or "20" or "21" or "22" or "23" or "24") adj (month* old or month* of age)).mp.
  6. ((one or two or three or four or five or six or seven or eight or nine or ten or eleven or twelve or thirteen or fourteen or fifteen or sixteen or seventeen or eighteen or nineteen or twenty or twenty-one or twenty-two or twenty-three or twenty-four) adj (month* old or month* of age)).mp.
  7. (age* adj ("1" or "2" or "3" or "4" or "5" or "6" or "7" or "8" or "9" or "10" or "11" or "12" or "13" or "14" or "15" or "16" or "17" or "18" or "19" or "20" or "21" or "22" or "23" or "24") adj month*).mp.
  8. (age* adj (one or two or three or four or five or six or seven or eight or nine or ten or eleven or twelve or thirteen or fourteen or fifteen or sixteen or seventeen or eighteen or nineteen or twenty or twenty-one or twenty-two or twenty-three or twenty-four) adj month*).mp.
  9. (infan* or neonat* or child* or pediatric* or paediatric*).jw.
  10. exp CHILD, PRESCHOOL/ or exp INFANT/ or exp Pediatrics/ or exp INFANT, NEWBORN/
  11. or/4-10
  12. Trimethoprim, sulfamethoxazole drug combination/
  13. (bactrim OR septrin OR septrim OR septra OR bactimel OR CPT OR cotrimoxazole OR co-trimoxazole OR trimethoprim-sulfamethoxazole OR sulfamethoxazole-trimethoprim).mp
  14. 12 or 13
  15. randomized controlled trial.pt.
  16. controlled clinical trial.pt.
  17. randomized.ab.
  18. placebo.ab.
  19. clinical trials as topic.sh.
  20. randomly.ab.
  21. trial.ti.
  22. 15 or 16 or 17 or 18 or 19 or 20 or 21
  23. exp animals/ not humans.sh.
  24. 22 not 23
  25. 3 and 11 and 14 and 24

**OvidSP EMBASE search**

1. (HIV or HIV-1 or HIV-2 or human immunodeficiency virus or human immune-deficiency virus or human immuno-deficiency virus or AIDS or Acquired Immunodeficiency Syndrome or Acquired Immune-Deficiency Syndrome or acquired immunedeficiency syndrome or acquired immuno-deficiency syndrome or HEU or PMTCT or mother-to-child transmission or vertical transmission or maternal-fetal transmission or maternal-foetal transmission or fetomaternal Infection or maternal-fetal infection or maternal-foetal infection or vertical infection).mp
2. exp Human immunodeficiency virus/ or exp Human immunodeficiency virus infection/
3. 1 or 2
4. (infan* or newborn* or new-born* or perinat* or neonat* or neo-nat* or preemie* or baby or babies or toddler* or child* or pediatric* or paediatric* or girl* or boy* or preschool* or pre-school*).mp
5. (("1" or "2" or "3" or "4" or "5" or "6" or "7" or "8" or "9" or "10" or "11" or "12" or "13" or "14" or "15" or "16" or "17" or "18" or "19" or "20" or "21" or "22" or "23" or "24") adj (month* old or month* of age)).mp.
6. ((one or two or three or four or five or six or seven or eight or nine or ten or eleven or twelve or thirteen or fourteen or fifteen or sixteen or seventeen or eighteen or nineteen or twenty or twenty-one or twenty-two or twenty-three or twenty-four) adj (month* old or month* of age)).mp.
7. (age* adj ("1" or "2" or "3" or "4" or "5" or "6" or "7" or "8" or "9" or "10" or "11" or "12" or "13" or "14" or "15" or "16" or "17" or "18" or "19" or "20" or "21" or "22" or "23" or "24") adj month*).mp.
8. (age* adj (one or two or three or four or five or six or seven or eight or nine or ten or eleven or twelve or thirteen or fourteen or fifteen or sixteen or seventeen or eighteen or nineteen or twenty or twenty-one or twenty-two or twenty-three or twenty-four) adj month*).mp.
9. (infan* or neonat* or child* or pediatric* or paediatric*).jw.
10. Exp preschool child/ or exp infant/ or exp pediatrics/ or exp toddler/
11. or/4-10
12. cotrimoxazole/
13. (bactrim OR septrin OR septrim OR septra OR bactimel OR CPT OR cotrimoxazole OR co-trimoxazole OR trimethoprim-sulfamethoxazole OR sulfamethoxazole-trimethoprim).mp
14. 12 or 13
15. Randomized controlled trial/
16. Controlled clinical study/
17. random$.ti,ab.
18. randomization/
19. intermethod comparison/
20. placebo.ti,ab.
21. (compare or compared or comparison).ti.
22. ((evaluated or evaluate or evaluating or assessed or assess) and (compare or compared or comparing or comparison)).ab.
23. (open adj label).ti,ab.
24. ((double or single or doubly or singly) adj (blind or blinded or blindly)).ti,ab.
25. double blind procedure/
26. parallel group$1.ti,ab.
27. (crossover or cross over).ti,ab.
28. ((assign$ or match or matched or allocation) adj5 (alternate or group$1 or intervention$1 or patient$1 or subject$1 or participant$1)).ti,ab.
29. (assigned or allocated).ti,ab.
30. (controlled adj7 (study or design or trial)).ti,ab.
31. (volunteer or volunteers).ti,ab.
32. human experiment/
33. trial.ti.
34. or/15-33
35. random$ adj sampl$ adj7 ("cross section$" or questionnaire$1 or survey$ or database$1)).ti,ab. not (comparative study/ or controlled study/ or randomi?ed controlled.ti,ab. or randomly assigned.ti,ab.)
36. Cross-sectional study/ not (randomized controlled trial/ or controlled clinical study/ or controlled study/ or randomi?ed controlled.ti,ab. or control group$1.ti,ab.)
37. (((case adj control$) and random$) not randomi?ed controlled).ti,ab.
38. (Systematic review not (trial or study)).ti.
39. (nonrandom$ not random$).ti,ab.
40. "Random field$".ti,ab.
41. (random cluster adj3 sampl$).ti,ab.
42. (review.ab. and review.pt.) not trial.ti.
43. "we searched".ab. and (review.ti. or review.pt.)
44. "update review".ab.
45. (databases adj4 searched).ab.
46. (rat or rats or mouse or mice or swine or porcine or murine or sheep or lambs or pigs or piglets or rabbit or rabbits or cat or cats or dog or dogs or cattle or bovine or monkey or monkeys or trout or marmoset$1).ti. and animal experiment/
47. Animal experiment/ not (human experiment/ or human/)
48. or/35-47
49. 34 not 48
50. 3 and 11 and 14 and 49

**Wiley Cochrane CENTRAL Register of Studies / Controlled Trials**

1. MeSH descriptor: [HIV] explode all trees
2. (hiv or "hiv-1" OR "hiv-2" OR "human immunodeficiency virus" OR "human immune-deficiency virus" OR "human immuno-deficiency virus" OR aids OR "Acquired Immunodeficiency Syndrome" OR "Acquired Immune-Deficiency Syndrome" OR "acquired immunedeficiency syndrome" OR "acquired immuno-deficiency syndrome" OR heu OR pmtct OR "mother-to-child transmission" OR "vertical transmission" OR "maternal-fetal transmission" OR "maternal-foetal transmission" OR "fetomaternal Infection" OR "maternal-fetal infection" OR "maternal-foetal infection" OR "vertical infection")
3. #1 or #2
4. MeSH descriptor: [Trimethoprim, Sulfamethoxazole Drug Combination] explode all trees
5. (bactrim OR septrin OR septrim OR septra OR bactimel OR CPT OR cotrimoxazole OR co-trimoxazole OR trimethoprim-sulfamethoxazole OR sulfamethoxazole-trimethoprim)
6. #4 or #5
7. MeSH descriptor: [Infant] explode all trees
8. MeSH descriptor: [Child] explode all trees
9. (infan* or newborn* or new-born* or perinat* or neonat* or neo-nat* or preemie* or baby or babies or toddler* or child* or pediatric* or paediatric* or girl* or boy* or preschool* or pre-school*)
10. (("1" or "2" or "3" or "4" or "5" or "6" or "7" or "8" or "9" or "10" or "11" or "12" or "13" or "14" or "15" or "16" or "17" or "18" or "19" or "20" or "21" or "22" or "23" or "24") adj (month* old or month* of age)).mp.
11. ((one or two or three or four or five or six or seven or eight or nine or ten or eleven or twelve or thirteen or fourteen or fifteen or sixteen or seventeen or eighteen or nineteen or twenty or twenty-one or twenty-two or twenty-three or twenty-four) adj (month* old or month* of age)).mp.
12. (age* adj ("1" or "2" or "3" or "4" or "5" or "6" or "7" or "8" or "9" or "10" or "11" or "12" or "13" or "14" or "15" or "16" or "17" or "18" or "19" or "20" or "21" or "22" or "23" or "24") adj month*).mp.
13. (age* adj (one or two or three or four or five or six or seven or eight or nine or ten or eleven or twelve or thirteen or fourteen or fifteen or sixteen or seventeen or eighteen or nineteen or twenty or twenty-one or twenty-two or twenty-three or twenty-four) adj month*).mp.
14. (infan* or neonat* or child* or pediatric* or paediatric*).jw.
15. #7 or #8 or #9 or #10 or #11 or #12 or #13 or #14
16. #3 and #6 and #15

We used the Cochrane RCT filter (3D as the review focus is co-trimoxazole prophylaxis, not treatment) to find clinically-focused RCTs.

**OvidSP Global Health**

1. (HIV or HIV-1 or HIV-2 or human immunodeficiency virus or human immune-deficiency virus or human immuno-deficiency virus or AIDS or Acquired Immunodeficiency Syndrome or Acquired Immune-Deficiency Syndrome or acquired immunedeficiency syndrome or acquired immuno-deficiency syndrome or HEU or PMTCT or mother-to-child transmission or vertical transmission or maternal-fetal transmission or maternal-foetal transmission or fetomaternal Infection or maternal-fetal infection or maternal-foetal infection or vertical infection).mp
2. exp human immunodeficiency viruses/
3. 1 or 2
4. (infan* or newborn* or new-born* or perinat* or neonat* or neo-nat* or preemie* or baby or babies or toddler* or child* or pediatric* or paediatric* or girl* or boy* or preschool* or pre-school*).mp
5. (("1" or "2" or "3" or "4" or "5" or "6" or "7" or "8" or "9" or "10" or "11" or "12" or "13" or "14" or "15" or "16" or "17" or "18" or "19" or "20" or "21" or "22" or "23" or "24") adj (month* old or month* of age)).mp.
6. ((one or two or three or four or five or six or seven or eight or nine or ten or eleven or twelve or thirteen or fourteen or fifteen or sixteen or seventeen or eighteen or nineteen or twenty or twenty-one or twenty-two or twenty-three or twenty-four) adj (month* old or month* of age)).mp.
7. (age* adj ("1" or "2" or "3" or "4" or "5" or "6" or "7" or "8" or "9" or "10" or "11" or "12" or "13" or "14" or "15" or "16" or "17" or "18" or "19" or "20" or "21" or "22" or "23" or "24") adj month*).mp.
8. (age* adj (one or two or three or four or five or six or seven or eight or nine or ten or eleven or twelve or thirteen or fourteen or fifteen or sixteen or seventeen or eighteen or nineteen or twenty or twenty-one or twenty-two or twenty-three or twenty-four) adj month*).mp.
9. (infan* or neonat* or child* or pediatric* or paediatric*).jw.
10. exp Infants/ or exp Neonates/ or exp preschool children
11. or/4-10
12. exp co-trimoxazole/
13. (bactrim OR septrin OR septrim OR septra OR bactimel OR CPT OR cotrimoxazole OR co-trimoxazole OR trimethoprim-sulfamethoxazole OR sulfamethoxazole-trimethoprim).mp
14. 12 or 13
15. randomized controlled trial.pt.
16. controlled clinical trial.pt.
17. randomized.ab.
18. placebo.ab.
19. clinical trials as topic.sh.
20. randomly.ab.
21. trial.ti.
22. 15 or 16 or 17 or 18 or 19 or 20 or 21
23. ((rat or rats or mouse or mice or swine or porcine or murine or sheep or lambs or pigs or piglets or rabbit or rabbits or cat or cats or dog or dogs or cattle or bovine or monkey or monkeys or trout or marmoset$1) not man).sh.
24. 22 not 23
25. 3 and 11 and 14 and 24

**Ebsco CINAHL Plus**

S1 MH randomized controlled trials

S2 MH double‐blind studies

S3 MH single‐blind studies

S4 MH random assignment

S5 MH pretest‐posttest design

S6 MH cluster sample

S7 TI (randomised OR randomized)

S8 AB (random*)

S9 TI (trial)

S10 MH (sample size) AND AB (assigned OR allocated OR control)

S11 MH (placebos)

S12 PT (randomized controlled trial)

S13 AB (control W5 group)

S14 MH (crossover design) OR MH (comparative studies)

S15 AB (cluster W3 RCT)

S16 MH animals+

S17 MH (animal studies)

S18 TI (animal model*)

S19 S16 OR S17 OR S18

S20 MH (human)

S21 S19 NOT S20

S22 S1 OR S2 OR S3 OR S4 OR S5 OR S6 OR S7 OR S8 OR S9 OR S10 OR S11 OR S12 OR S13 OR S14 OR S15

S23 S22 NOT S21

S24 (hiv OR "hiv-1" OR "hiv-2" OR "human immunodeficiency virus" OR "human immune-deficiency virus" OR "human immuno-deficiency virus" OR aids OR "Acquired Immunodeficiency Syndrome" OR "Acquired Immune-Deficiency Syndrome" OR "acquired immunedeficiency syndrome" OR "acquired immuno-deficiency syndrome" OR heu OR pmtct OR "mother-to-child transmission" OR "vertical transmission" OR "maternal-fetal transmission" OR "maternal-foetal transmission" OR "fetomaternal Infection" OR "maternal-fetal infection" OR "maternal-foetal infection" OR "vertical infection")

S25 (MH "Human Immunodeficiency Virus+")  OR (MH "Acquired Immunodeficiency Syndrome+") OR (MH "HIV Seropositivity+") OR (MH "HIV-Infected Patients+")

S26: S24 OR S25

S27 (bactrim OR septrin OR septrim OR septra OR bactimel OR CPT OR cotrimoxazole OR co-trimoxazole OR trimethoprim-sulfamethoxazole OR sulfamethoxazole-trimethoprim)

S28 (MH "Trimethoprim-Sulfamethoxazole Combination")

S29: S27 OR S28

S30 (infan* or newborn* or new-born* or perinat* or neonat* or neo-nat* or preemie* or baby or babies or toddler* or child* or pediatric* or paediatric* or girl* or boy* or preschool* or pre-school*)

S31 (MH "Infant+") OR (MH "Child+")

S32: S30 OR S31

S33: S26 AND S29 AND S32

S34: S33 AND 23

**Ebsco Africa-Wide Information**

S1 ("hiv-1" OR "hiv-2" OR "human immunodeficiency virus" OR "human immune-deficiency virus" OR "human immuno-deficiency virus" OR aids OR "Acquired Immunodeficiency Syndrome" OR "Acquired Immune-Deficiency Syndrome" OR "acquired immunedeficiency syndrome" OR "acquired immuno-deficiency syndrome" OR heu OR pmtct OR "mother-to-child transmission" OR "vertical transmission" OR "maternal-fetal transmission" OR "maternal-foetal transmission" OR "fetomaternal Infection" OR "maternal-fetal infection" OR "maternal-foetal infection" OR "vertical infection")

S2 (bactrim OR septrin OR septrim OR septra OR bactimel OR CPT OR cotrimoxazole OR co-trimoxazole OR trimethoprim-sulfamethoxazole OR sulfamethoxazole-trimethoprim)

S3 (infan* or newborn* or new-born* or perinat* or neonat* or neo-nat* or preemie* or baby or babies or toddler* or child* or pediatric* or paediatric* or girl* or boy* or preschool* or pre-school*)

S1 AND S2 AND S3

**SciELO**

tw:((hiv OR "hiv-1" OR "hiv-2" OR "human immunodeficiency virus" OR "human immune-deficiency virus" OR "human immuno-deficiency virus" OR aids OR "Acquired Immunodeficiency Syndrome" OR "Acquired Immune-Deficiency Syndrome" OR "acquired immunedeficiency syndrome" OR "acquired immuno-deficiency syndrome" OR heu OR pmtct OR "mother-to-child transmission" OR "vertical transmission" OR "maternal-fetal transmission" OR "maternal-foetal transmission" OR "fetomaternal Infection" OR "maternal-fetal infection" OR "maternal-foetal infection" OR "vertical infection") AND (bactrim OR septrin OR septrim OR septra OR bactimel OR cpt OR cotrimoxazole OR co-trimoxazole OR trimethoprim-sulfamethoxazole OR sulfamethoxazole-trimethoprim) AND (infan* OR newborn* OR new-born* OR perinat* OR neonat* OR neo-nat* OR preemie* OR baby OR babies OR toddler* OR child* OR pediatric* OR paediatric* OR girl* OR boy* OR preschool* OR pre-school*))

**WHO Global Index Medicus**

tw:((hiv OR "hiv-1" OR "hiv-2" OR "human immunodeficiency virus" OR "human immune-deficiency virus" OR "human immuno-deficiency virus" OR aids OR "Acquired Immunodeficiency Syndrome" OR "Acquired Immune-Deficiency Syndrome" OR "acquired immunedeficiency syndrome" OR "acquired immuno-deficiency syndrome" OR heu OR pmtct OR "mother-to-child transmission" OR "vertical transmission" OR "maternal-fetal transmission" OR "maternal-foetal transmission" OR "fetomaternal Infection" OR "maternal-fetal infection" OR "maternal-foetal infection" OR "vertical infection")

AND

(bactrim OR septrin OR septrim OR septra OR bactimel OR cpt OR cotrimoxazole OR co-trimoxazole OR trimethoprim-sulfamethoxazole OR sulfamethoxazole-trimethoprim)

AND

(infan* OR newborn* OR new-born* OR perinat* OR neonat* OR neo-nat* OR preemie* OR baby OR babies OR toddler* OR child* OR pediatric* OR paediatric* OR girl* OR boy* OR preschool* OR pre-school*))

**https://www.clinicaltrials.gov/**

HIV AND (Trimethoprim, Sulfamethoxazole OR co-trimoxazole)

Child (Birth-17)

**https://www.who.int/clinical-trials-registry-platform**

HIV AND co-trimoxazole OR HIV AND Trimethoprim, Sulfamethoxazole

Children only

**S3 Appendix: Definition of children who are HIV-exposed and uninfected**

Children born to women living with HIV (HIV-exposed) may become infected through intrauterine, intrapartum or postnatal (breastmilk) transmission. The risk of HIV transmission therefore remains until cessation of breastfeeding, after which time the child may be confirmed to be uninfected. The diagnosis of HIV relies on nucleic acid testing (NAT) in the first 18 months after birth. This is because maternal HIV antibodies remain in the child’s circulation after birth and therefore only reflect HIV exposure during this period. Infants who are HEU refer to HIV-exposed infants with a negative NAT or antibody test. The majority of countries test for infant HIV at 4-6 weeks of age; however, HIV-exposed children remain at risk of infection throughout the postnatal period due to breastfeeding, therefore true CHEU status is only established after full cessation of breastfeeding. For the purpose of this review, inclusion criteria will include HEU status confirmation following the DECIPHER guidelines (*Burmen B., The DECIPHER Project. 2019*) for high certainty that the child is HEU, given below:

Child of mother known to be living with HIV

AND

Child *tested HIV negative* under at least one of the following scenarios

1. At the end of study period under investigation or later [no breastfeeding or ARV prophylaxis information required]
2. At the time of measurement of study outcome of interest or later [no breastfeeding or ARV prophylaxis information required]
3. In the absence of breastfeeding and infant/child ARV prophylaxis, child tested HIV negative at least once ≥ 6 weeks of age
4. If child was breastfed without extended child ARV prophylaxis, child tested HIV negative ≥ 6 weeks after end of breastfeeding
5. If child received ARV prophylaxis but was never breastfed, child tested HIV negative ≥ 4 weeks after ARV prophylaxis completion AND > 6 weeks of age
6. If child was breastfed with extended child ARV prophylaxis, child tested HIV negative ≥ 6 weeks after end of breastfeeding AND > 4 weeks after ARV prophylaxis completion

**S4 Appendix**

**Characteristics of excluded articles and trial summaries on full text (ordered by first author and year) (n=60)**

| Study/Trial | Reason for exclusion |
| --- | --- |
| Aizire et al, 2012 | Did not meet comparator criteria (no controls) |
| Ali et al, 2020 | Did not meet publication criteria (conference abstract) |
| Aliyu et al, 2016 | Did not meet intervention criteria (intervention not specific to co-trimoxazole) |
| Berkley et al, 2016 | Did not meet population criteria (not focused on children who are HEU) |
| Bigira 2011 | Did not meet publication criteria (conference abstract)  Full-text checked |
| Boettiger et al, 2018 | Did not meet publication criteria (conference abstract)  *Full-text below* |
| Boettiger et al, 2019 | Did not meet population criteria (children with HIV infection) |
| Bork et al, 2013 | Did not meet publication criteria (conference abstract)  *Full-text below* |
| Bork et al, 2014 | Did not meet comparator criteria (no controls) |
| Chokephaibulkit et al, 1999 | Did not meet population criteria (children with HIV infection) |
| Chokephaibulkit et al, 2000 | Did not meet comparator criteria (no controls) |
| Coutsoudis et al, 2011 | Did not meet study design criteria (not an RCT) |
| Coutsoudis et al, 2016 | Did not meet outcome criteria (protocol only) |
| Davis et al, 2015 | Did not meet study design criteria (not an RCT) |
| Davis et al, 2016 | Did not meet publication criteria (conference abstract)  *Full-text below* |
| Davis et al, 2017 | Did not meet study design criteria (not an RCT) |
| Doczeova et al, 2005 | Did not meet population criteria (children with HIV infection) |
| Dow et al, 2012 | Did not meet study design criteria (not an RCT) |
| Dryden-Peterson et al, 2011 | Did not meet publication criteria (conference abstract)  *Full-text below* |
| Dryden-Peterson et al, 2013 | Did not meet study design criteria (not an RCT) |
| Duke, 2011 | Did not meet study design criteria (commentary) |
| Ewing et al, 2017a | Did not meet study design criteria (not an RCT) |
| Ewing et al, 2017b | Did not meet publication criteria (conference abstract)  *Full-text above* |
| Ewing et al, 2019 | Did not meet study design criteria (not an RCT) |
| Farley et al, 1994 | Did not meet intervention criteria (no co-trimoxazole intervention) |
| Field Exchange Emergency Nutrition Network, 2016 | Did not meet population criteria (not focused on children who were HEU) |
| Gill et al, 2003 | Did not meet study design criteria (letter) |
| Graham et al, 2004 | Did not meet study design criteria (commentary) |
| Green et al, 2007 | Did not meet study design criteria (systematic review) |
| Green et al, 2007 | Did not meet study design criteria (systematic review) |
| Grimwade et al, 2003 | Did not meet study design criteria (systematic review) |
| Grimwade et al, 2006 | Did not meet study design criteria (systematic review) |
| Hobbs et al, 2012 | Did not meet study design criteria (not humans) |
| Hobbs et al, 2017 | Did not meet study design criteria (not humans) |
| Homsy et al, 2014a | Did not meet publication criteria (conference abstract)  *Full-text included* |
| Humphreys et al, 2010 | Did not meet study design criteria (systematic review) |
| Kakaru et al, 2010 | Did not meet publication criteria (conference abstract)  *Full-text below* |
| Kakaru, 2011 | Did not meet publication criteria (conference abstract)  *Full-text below* |
| Kakaru et al, 2013 | Did not meet outcome criteria (gametocytemia) |
| Kinara et al, 2013 | Did not meet publication criteria (conference abstract)  *Full-text included* |
| Leach-Lemens, 2012 | Did not meet comparator criteria (no controls) |
| Manyando et al, 2013 | Did not meet study design criteria (systematic review) |
| Mbeye et al, 2014 | Did not meet study design criteria (systematic review) |
| Onakpoya et al, 2015 | Did not meet study design criteria (systematic review) |
| Powis et al, 2016 | Did not meet publication criteria (conference abstract)  *Full-text included* |
| Sandison et al, 2009 | Did not meet publication criteria (conference abstract)  *Full-text included* |
| Shahid, 2008 | Did not meet study design criteria (letter) |
| Shapiro et al, 2016 | Did not meet publication criteria (conference abstract)  *Full-text included* |
| Sibanda et al, 2011 | Did not meet study design criteria (systematic review) |
| Thera et al, 2005 | Did not meet population criteria (not focused on children who are HEU) |
| Tumwebaze et al, 2015 | Did not meet population criteria (combined children who are HEU and children with HIV) |
| *Trial register reports* |  |
| NCT0093442 | Did not meet population criteria (not focused on children who are HEU) |
| NCT02282293 | Did not meet comparator criteria (both arms got co-trimoxazole) |
| NCT01086878 | Did not meet study design criteria (not RCT) |
| NCT03517878 | Did not meet study design criteria (not RCT) |
| NCT02094508* | Did not meet outcome criteria (no outcomes reported) |
| NCT00527800^†^ | Duplicate trials of full papers that are already included; did not meet outcome criteria (no data) |
| NCT01229761^†^ | Duplicate trials of full papers that are already included; did not meet outcome criteria (no data) |
| NCT00948896^†^ | Duplicate trials of full papers that are already included; did not meet outcome criteria (no data) |
| PACTR201311000621110^†^ | Duplicate trials of full papers that are already included; did not meet outcome criteria (no data) |

* This trial fitted all the inclusion criteria, however, it was stopped early for futility and no relevant study outcomes were obtained.

^†^ These are reports from the register of studies that are duplicates of full papers that are included. These reports contain no data and have been excluded.

**S5 Appendix: Details of included trials**

| Trial | Report |
| --- | --- |
| RCT of effect of cotrimoxazole on the health of HIV-exposed uninfected infants (South Africa)  PACTR201311000621110 | Daniels et al, 2019  (Main study results) |
|  | D’Souza et al, 2020  (Antimicrobial resistance results) |
| Study to Improve Survival Among HIV-Exposed Infants in Botswana (Mpepu) (Botswana)  NCT01229761 | Lockman et al, 2017  (main study results) |
|  | Powis et al, 2017  (Antimicrobial resistance results) |
| Interactions Between HIV and Malaria in African Children (TCC) (Uganda)  NCT00527800 | Sandison et al, 2011  (Early study results) |
|  | Homsy et al, 2014  (Later study results) |
| Chemopreventive Therapy for Malaria in Ugandan Children (PROMOTE-Chemop)  (Uganda)  NCT00948896 | Kamya et al, 2014  (Study results) |

**S6 Appendix: Risk of bias assessments**

|  | Selection bias | Performance bias | Attrition bias | Detection bias | Reporting bias | Other | Overall |
| --- | --- | --- | --- | --- | --- | --- | --- |
|  | *Domain 1: Risk of bias arising from the randomization process* | *Domain 2: Risk of bias due to deviations from the intended interventions* | *Domain 3: Missing outcome data* | *Domain 4: Risk of bias in measurement of the outcome* | *Domain 5: Risk of bias in selection of the reported result* | *Comments* | *Overall risk of bias* |
|  |  |  |  |  |  |  |  |
| Daniels et al, 2019 | Low risk | Low risk | High risk ^a^ | Low risk | Low risk | Single blinded | High risk |
| Lockman et al, 2017 | Low risk | Low risk | Some concerns ^b^ | Low risk | Low risk | Blinded | Some concerns |
| Sandison et al, 2011 | Low risk | Low risk | Low risk | Some concerns ^e^ | Low risk | Not blinded | Some concerns |
| Homsy et al, 2014 | Some concerns ^c^ | Low risk | Some concerns ^d^ | Some concerns ^e^ | Low risk | Not blinded | High risk |
| Kamya et al, 2014 | Low risk | Low risk | Low risk | Some concerns ^e^ | Some concerns ^f^ | Not blinded | Some concerns |

**Footnotes:** Risk of bias was assessed using the Cochrane Collaboration’s Risk of Bias 2.0 tool (https://www.riskofbias.info/welcome/rob-2-0-tool/current-version-of-rob-2)

Risk of bias assessments presented as in relation to mortality outcome. Results are similar for infectious morbidity outcomes.

^a^ 15% loss to follow-up (183/1219), 7% relocated, 8% censored when study closed. Of those lost to follow up, missingness could have depended on its true value. No further information available.

^b^ 5% loss to follow-up (153/2828), 39% censored due to DSMB. Loss to follow-up could have depended on its true value, but small percentage. No difference in timing of censorship between groups.

^c^ Malaria incidence before second randomization was lower in those children assigned to continue co-trimoxazole from 2 years, compared to those stopping co-trimoxazole (IRR = 1.48, p=0.06)

^d^ Overall 11% missing data: 1% loss to follow-up, 5% relocated, 2% withdrew informed consent, 2% unable to comply with protocol or tolerate drugs

^e^ Outcome assessors were aware of the intervention received by study participants; assessment of outcome could have been influenced by knowledge of the intervention received, eg mothers in control group may have been more likely to have attended clinics

^f^ No information on a pre-specified analysis plan.

**S7 Appendix: Neutropenia and anaemia in trials examining the effects of co-trimoxazole prophylaxis on children who are HEU**

| Study | Neutropenia | | Anaemia | |
| --- | --- | --- | --- | --- |
|  | **Co-trimoxazole** | **No co-trimoxazole** | **Co-trimoxazole** | **No co-trimoxazole** |
| Daniels et al, 2019 | 1 (1.5%) | 0% | 4 (6.0%) | 6 (8.8%) |
|  | p=0.649 | | p=0.089 | |
| Lockman et al, 2017 | 95 (8.1%) | 68 (5.8%) | 83 (8.1%) | 81 (8.3%) |
|  | Difference: -2.3% (-0.2 to 4.3), p=0.03 | | Difference: -0.1% (-2.8 to 2.6), p=0.93 | |
| Sandison et al, 2011 | NR | NR | NR | NR |
|  | Reported no difference | | Reported no difference | |
| Homsy et al, 2014 | 9 (0.048 ppy) | 10 (0.057 ppy) | 51 (0.272 ppy) | 67 (0.379 ppy) |
|  | IRR: 0.85 (0.34 to 2.15), p=0.73 | | IRR: 0.69 (0.34 to 1.40), p=0.31 | |
| Kamya et al, 2014 | - | - | 22/285  (7.7%) | 42/362  (11.6%) |
|  |  |  | Protective efficacy: 7% (-100 to 57), p=0.86;  Grade 3-4: p=0.009 | |

**Footnote:**

Daniels *et al* report that routine blood testing was stopped after the first 100 participants were randomly assigned following permission from the DSMB and ethics committee, therefore the *N* is lower for these outcomes compared to the full trial. Proportions given are Grade 3 and 4 results at 12 months, p-values calculated across all grades at 12 months. Lockman *et al* report estimated proportions. Sandison *et al:* Results in anaemia column represent median haemoglobin concentration at day 0 of malaria episodes or change at follow-up. Abbreviations: NR: Not reported; ppy: incidence per person year at risk.
